# First-person disavowals of digital phenotyping and epistemic injustice in psychiatry

**DOI:** 10.1007/s11019-023-10174-8

**Published:** 2023-09-19

**Authors:** Stephanie K. Slack, Linda Barclay

**Affiliations:** Philosophy, School of Philosophical, Historical and International Studies, Monash University, Clayton, VIC 3800 Australia

**Keywords:** Digital phenotyping, Mental health, Epistemic injustice, Ethics, Psychiatry

## Abstract

Digital phenotyping will potentially enable earlier detection and prediction of mental illness by monitoring human interaction with and through digital devices. Notwithstanding its promises, it is certain that a person’s digital phenotype will at times be at odds with their first-person testimony of their psychological states. In this paper, we argue that there are features of digital phenotyping in the context of psychiatry which have the potential to exacerbate the tendency to dismiss patients’ testimony and treatment preferences, which can be instances of epistemic injustice. We first explain what epistemic injustice is, and why it is argued to be an extensive problem in health and disability settings. We then explain why epistemic injustice is more likely to apply with even greater force in psychiatric contexts, and especially where digital phenotyping may be involved. Finally, we offer some tentative suggestions of how epistemic injustice can be minimised in digital psychiatry.

## Introduction

Digital phenotyping has been hailed as a promising new tool which will potentially enable detection and diagnosis of mental illness (Martinez-Martin et al. 2018). By monitoring human interaction with and through digital devices, such as wearables or smartphones, digital phenotyping could be used to predict risk or relapse of mental illness, such as depression or psychosis. It is possible this may lead to earlier intervention and treatment and improve mental health outcomes (Insel 2017).

Notwithstanding its promises, it is certain that a person’s digital phenotype will at times be at odds with their first-person testimony of their psychological states. Digital phenotyping may predict a person is experiencing depression which the person denies. Such a situation raises special ethical concerns in the context of psychiatry. A common response to patients of psychiatry when they disagree with clinical assessments or treatment recommendations is to suggest their resistance is part of the mental illness (cf. Coghlan and D’Alfonso 2021). Further, this can lead to a complete disregard of patients’ treatment preferences when they are subject to involuntary treatment orders (Gustafsson et al. 2014; Nyttingnes et al. 2016; Ridley and Hunter 2013).

In this paper, we argue that there are features of digital phenotyping in the context of psychiatry which have the potential to exacerbate the tendency to dismiss patients’ testimony and treatment preferences. Many (although not all) such instances of such interactions between clinicians and patients are cases involving epistemic injustice, where people with mental illness experience an injustice in relation to their status as knowing agents. In developing this argument, we first explain what epistemic injustice is, and why it is argued to be an extensive problem in health and disability settings. We then argue that the features of health and disability care that make epistemic injustice more likely apply with even greater force in psychiatric contexts, and especially where digital phenotyping may be involved.

Drawing attention to how epistemic injustice is enacted in psychiatry, and could be replicated when using digital phenotyping, is important given that digital phenotyping could be used as basis for treatment intervention, sometimes involuntary intervention. In doing so, we do not seek to settle the question of which perspective ought to be regarded as authoritative where disagreements arise; of course, this will require a complex balancing of evidence and ethical trade-offs relevant to the situation and individual. Rather, we suggest we should be cautious with regard to our enthusiasm for digital phenotyping given its potential to perpetuate and exacerbate existing practices of epistemic injustice in psychiatric practice. Many of the issues we raise may apply more broadly to clinical applications of artificial intelligence (AI) and machine learning for predictive analytics, including outside of psychiatry. However, our discussion focuses on digital phenotyping in psychiatry, both to focus the discussion and because features of both epistemic injustice and digital phenotyping in this context raise particular concerns.

The paper proceeds as follows. In the next section we provide a brief overview of what digital phenotyping is, and the promise it holds for the early detection and diagnosis of mental illness. In Sect. 3 we discuss familiar forms of epistemic injustice and explain why such injustice is common in health and disability settings. In Sect. 4 we explain why people with a history of psychiatric illness are particularly vulnerable to epistemic injustice and why digital phenotyping is likely to increase instances of such injustice. In Sect. 5 we offer some tentative suggestions for how epistemic injustice can be minimised in digital psychiatry.

## Digital phenotyping in psychiatry

One does not always find consistent use of the term ‘digital phenotyping’ within the literature. For our purposes, we use digital phenotyping to mean the process of continuously collecting and analysing digital data derived from human interaction with digital products to make assessment or inferences about illness (Jain et al. 2015). Digital phenotyping can include, but is not limited to, sensors and electronic activities like GPS, phone calls, barometers, light sensors, accelerometers and voice and text capture, gestural sensing, email use, web browsing and interaction with screens (Coghlan and D’Alfonso 2021). Such digital sensing can be passive or more active, such as users responding to prompts for data. It can also be what Simon Coghlan and Simon D’Alfonso call interactive (2021, 1908) which refers to users or wearers of devices swiping, tapping, talking and so on. The resulting aggregation of unstructured data – the digital exhaust, or footprint (Coghlan and D’Alfonso 2021, 1909) - can be analysed using machine learning algorithms to identify patterns in the data. In psychiatry, digital phenotyping is specifically concerned with using this digital data to make inferences about the risk of, or presence of, mental illness such as depression or schizophrenia.

As one example of how digital phenotyping could be used in psychiatric treatment, it is possible digital phenotyping has the potential to monitor and predict relapse of schizophrenia. A study using a digital phenotyping device to collect heart-rate variability, electrodermal activity and GPS movement of thirty people with schizophrenia and 25 controls, found that people with schizophrenia had lower levels or heart-rate variability and movement (Cella et al. 2018). The researchers suggest that these findings may be applicable to develop devices for the monitoring of wellbeing and relapse prevention in people with schizophrenia. Where lower levels of heart-rate variability and movement are detected, it may be inferred that a person is at risk of a relapse.

Much has been made about the potential for digital phenotyping to provide more personalised and objective mental health diagnoses. Nicole Martinez-Martin and colleagues argue that: “For psychiatry, which has heretofore relied exclusively on episodic reports of mood, digital phenotyping offers a powerful approach for the systematic detection of behavioral states, subtyping current heterogeneous diagnostic categories, and measuring outcomes” (Martinez-Martin et al. 2018, 1). The WPA-Lancet Commission on the future of psychiatry claims that digital phenotyping could provide more “objective behavioural data” than self-reports, the idea being that the continuous real-time collection of behavioural data and physiological information (such as heart rate, body temperature) can capture a range of information that could be used in the diagnosis of mental illness (Bhugra et al. 2017). Insel (2017) also suggests digital phenotyping offers the possibility of a more objective, measurement-based approach to diagnosing mental illness.

The underlying assumption across the framing of digital phenotyping seems to be that the data collected through digital sensing has the potential to be more objective than current methods of diagnosing mental illness, and more objective than patients’ reports of their own symptoms or activities. Indeed, sometimes this is explicitly stated in the literature: “The *objectivity* and seamless provision of information over a period of time brings advantages compared to subjective questionnaires at a certain point in time” (Winkler et al. 2022, 2, emphasis ours); and “This promising new approach has been developed as an *objective*, passive assessment tool for the diagnosis and treatment of mental illness” (Martinez-Martin et al. 2018, 1, emphasis ours).

Most of these claims about the potential of digital phenotyping to detect mental illness are at this stage largely promissory. As yet, there are few clinically validated digital sensing tools for the detection and diagnosis of mental illness (Torous et al. 2021), although it seems more likely than not that such tools will exist in the future. One obvious hurdle is to establish that the relevant relation exists between the ‘digital exhaust’ and a person’s psychological properties (“human traits, moods, behaviors, states, attitudes, orientations, feelings, conditions, and illnesses” (Coghlan and D’Alfonso 2021, 1911), namely, that the psychological properties are causing the digital data. This would be a more robust relation for the purposes of detecting and diagnosing mental illness than mere correlation or instances where it is the person’s interaction with digital sensing devices which is itself causing various psychological states of concern (Coghlan and D’Alfonso 2021).

To illustrate, Coghlan and D’Alfonso (2021) use the example of digital phenotyping detecting the presence of depression from passively monitoring the low level of battery charge on a person’s phone. Digital phenotyping detects a pattern of the phone battery not being charged and then an inference is made that depression is causing the individual to not charge their phone battery. These inferences are probabilistic: “since the strength of the inference depends on the presence and extent of the causal connection and the weight and accuracy of the data and the information features associated with the data-and these may not be known with certainty” (Coghlan and D’Alfonso 2021, 1912). It is possible there are other causes for a phone not being charged, such as laziness or perhaps the phone is switched off. “The aim in making this type of inference is to collect as much relevant information as possible to support a stronger inference from the data/information feature(s) to the psychological property that caused them” (Coghlan and D’Alfonso 2021, 1911).

Current exploratory studies on digital phenotyping do rely on collecting multiple information features taken together to support the inference that the relevant data is caused by psychological states associated with mental illness. While studies may have small sample sizes, recent research has shown that digital phenotyping may be useful in predicting relapse in schizophrenia. As one example, a study by Philip Henson and colleagues used smartphone digital phenotyping to capture a range of active and passive data including self-reported surveys, mobility from GPS data, sociability from call and text logs, cognition, screen time and sleep over a period of three to six months in 63 participants with schizophrenia and 27 controls (Henson et al. 2021). The study used ‘anomaly detection’ where all the data collected for each individual was analysed to identify a baseline for that individual and to identify anomalies. Anomalies were defined as: “days where given features deviated significantly from that baseline” (Henson et al. 2021, 3). In other words, on days where behaviours such as locations visited or the number and / or length of calls sent and received differ to usual patterns, it would be considered an anomaly. The Henson et al. (2021) study reported that it had 89% sensitivity and 75% specificity for predicting relapse in schizophrenia. This indicates that anomaly detection may be useful for predicting relapse in schizophrenia. This builds on previous research that has suggested that anomalies related to mobility, sociability and self-reported surveys are higher during the two weeks leading up to a relapse than at other times. However, it is important to note that Henson et al.’s study found that anomaly rates were significantly higher in the *passive* data collection of mobility and sociability compared to the active data: the self-reported surveys. As Henson et al. (2021) point out, more research is needed to improve specificity and understand how these tools could be used in clinical practice.

There will inevitably be many cases where such inferences from passive data are incorrect or contested. For example, Henson and colleagues acknowledge that: “smartphones are a proxy for behavior and do not represent the full context of someone’s environment. For example, a phone left on a table for several hours may be incorrectly interpreted as inactivity or sleep” (Henson et al. 2021, 5). Or consider where anomalies around the mobility and sociability features identified in the study discussed by Henson et al. (2021) are detected following a person’s discharge from hospital for a broken leg and a subsequent period of reduced activity and socialisation. Simple scenarios such as these indicate that there will be cases where the digital data is failing to accurately detect the presence of the relevant psychological states. Coghlan and D’Alfonso (2021) suggest additional methods for affirming inferences in such cases. For example, additional data can be secured, such as blood tests, or one may simply seek feedback from the person concerned (who can, for example, explain that they left their smartphone on the table or that they have broken their leg).

In what follows, we will argue that that the various options for scrutinising and challenging inferences from the data will be particularly vexed in the psychiatric context. Epistemic injustice in psychiatry makes it particularly likely that inferences of mental illness from digital sensing will evade robust levels of challenge and scrutiny, exposing psychiatric patients to the possibility of injustice and substandard medical care.

## Epistemic injustice in medicine

In order to develop our argument, we begin with a discussion of epistemic injustice and its presence in medicine.

Testimonial injustice refers to a speaker being afforded a deflated level of credibility by the hearer as a result of the hearer’s overt or unconscious prejudice against the social group to which the speaker belongs. In the paradigm cases outlined by Fricker (2007), the hearer deflates the credibility of the speaker’s testimony because of prejudice against the speaker’s sex or race. Such testimonial injustice can occur in medical settings. For example, David Peña-Guzmán and Joel Reynolds argue that a doctor can hold a group-based belief that black people have higher pain thresholds than people who are not black. That leads to testimonial injustice against their black patients when they discount the validity of their testimony concerning the amount of pain they are suffering (2019, 217).

Sex and race are not the only features of persons that can cause prejudice concerning their testimonial reliability: testimonial injustice is something experienced by individual members of a wider range of marginalised social groups, including within medicalised contexts. For example, it has been more recently argued that disabled people are often victims of testimonial injustice, a credibility deficit undergirded by the prejudice that to be disabled is to be a tragic, pitiable figure. It has been argued that the ‘disability paradox’ is evidence of testimonial injustice (Scully 2019). Empirical evidence suggests that when asked to rate their quality of life, disabled people often report a level only slightly below that reported by non-disabled people. Despite robust testimony from disabled people about the quality of their lives, nondisabled people rate disabled people’s quality of life much lower than disabled people do, with disparities explained away by reference to phenomena like sour grapes or adaptative preferences, or an inability to imagine something better (Scully 2019). In the medical context, healthcare professionals also often rate disabled people’s quality of life lower than disabled people themselves do: the testimony disabled people provide about their quality of life is not believed (Peña-Guzmán and Reynolds 2019). Similarly, Peña-Guzmán and Reynolds (2019) describe how a disabled person can suffer an arbitrary credibility deficit when a clinician discounts their testimony about why they have sought medical advice: someone with a mobility impairment enters a clinic with a rash which they believe is caused by an allergic reaction but the clinician ignores their testimony and instead assumes it is the wheelchair rubbing up against their skin that has caused the rash. ‘Hyper-attentiveness’ to a person’s disability can cause a clinician to discount the relevance or credibility of the patient’s testimony, behaviour on the part of clinicians which Peña-Guzman and Reynolds argue plays a significant role in medical errors (2019, 223-5).

Compounding the problem of testimonial injustice is what Peña-Guzmán and Reynolds have dubbed epistemic overconfidence (2019). In contrast to certain patient groups, medical professionals are often afforded a credibility *excess.* Clinicians can internalise this epistemic privilege and become so self-assured of their knowledge that they may often fail to consider an alternative diagnosis, seek a second opinion, critically reflect on non-medical determinants of illness, or revise their assumptions about disability. This can exacerbate the problem of discounting patient testimony. For example, epilepsy is frequently misdiagnosed in people with intellectual disability because clinicians cannot tell the difference between epileptic events and non-epileptic self-stimulatory events (Peña-Guzmán and Reynolds 2019). Peña-Guzmán and Reynolds suggest the problem here is that clinicians do not know that they cannot tell the difference and so jump to a diagnosis because of their overconfidence in their own medical knowledge.

Hermeneutical injustice is another form of epistemic injustice articulated by Fricker. On Fricker’s (2007) account, hermeneutical injustice occurs where structural prejudice means there is a gap in the collective hermeneutical resources, and this gap prevents a person from making sense of their experience. A person’s ability to make sense of the world and communicate with others requires a set of epistemic tools - narratives, concepts, associations, and the like. These are shared resources, which arise through shared practices and communication. A key example of hermeneutical injustice outlined by Fricker (2007) concerns sexual harassment. The fact of sexual harassment existed long before the concept of ‘sexual harassment’ entered our collective hermeneutical resources. This left women who experienced sexual harassment unable to understand or articulate their experiences adequately. Unequal social power explains the gap in hermeneutical resources. Different social groups enjoy different levels of social power and privilege, which in turn shapes the development of our collective hermeneutical resources – how features of the world are understood and explained. In particular, the concepts, vocabulary, and narratives that are particularly salient to the experiences of certain social groups are supressed or neglected because of the marginalisation of that social group. ‘Flirting’ became ‘sexual harassment’ only when women began to collectively articulate a more apt conception of the behaviour, something that, in turn, followed from larger numbers of women entering the workforce and holding positions of authority.

Hermeneutical injustice involves not only an absence of adequate hermeneutical resources. Marginalised social groups often do have rich hermeneutical resources to understand their own experiences. Here, the injustice consists in the fact that their alternative ways of understanding their own experiences and making sense of the world are excluded from more broadly shared understandings (Dotson 2012; Pohlhaus 2012). The marginalised group is therefore prevented from contributing to, and updating, dominant shared epistemic resources. Collective epistemic resources continue to be structurally prejudiced because they largely reflect the experiences and interpretations of dominant social groups. Given that marginalised hermeneutical resources are not more generally available, experiences and perspectives articulated by marginalised individuals can be unintelligible to others, or easily dismissed as nonsense (Scully 2019, 304). However, it is often reasonable to expect members of dominant epistemic communities to recognise their epistemic limitations and act accordingly. The dominant community’s ongoing failure to properly engage with marginalised epistemic resources is what Gaile Pohlhaus (2012) calls wilful hermeneutical injustice or Kristie Dotson (2012) contributory injustice. Consider the knowledge black people might have about police abuse of power, or the knowledge wheelchair users possess about how wheelchairs navigate physical spaces. Exclusion or dismissal of such knowledge is often willful, and has significant consequences for the lives of marginalised groups in terms of the relations to police or their ability to access public spaces.

Numerous scholars have argued that hermeneutical injustice is common in the medical context. Certain knowledge is prioritised and validated, such as medicalised ‘objective’ information at the expense of the subjective lived experience of the patient (Buchman et al. 2017; Kidd and Carel 2017; McCradden et al. 2023; Peña-Guzmán and Reynolds 2019). Daniel Buchman and colleagues outline how this occurs in the treatment of chronic pain whereby western medicine prioritises objective or scientific evidence such as X-Rays or MRIs to affirm the existence of the pain over the subjective testimony of patients experiencing the pain (Buchman et al. 2017). Healthcare systems, and the patient-clinician encounter is structured in such a way as to limit the types of information that are considered relevant and to privilege medicalised information. When patient testimony is sought, it is solicited so as to fit within the confines of the narrowly accepted framework of the medicalised information that the clinician views as important. Ian Kidd and Havi Carel argue ill people are vulnerable to such epistemic injustice whereby patient contributions can be dismissed because patients are judged to lack a sense of relevance to what is considered medically important; a patient may be deeply concerned about incontinence, or bodily estrangement, but this may be considered medically minor in the clinician’s diagnosis or treatment plan (Kidd and Carel 2017). They argue such experiences are labelled as subjective and so do not make a significantly meaningful impact on the practice of healthcare. This injustice is a form of contributory injustice when there is a wider refusal to consider and accept that the types of information provided by patients (their phenomenological experiences of illness) may be important knowledge that can contribute to better healthcare practice (Kidd and Carel 2017).

## Epistemic injustice, digital phenotyping, and psychiatry

We suggest that people with a history of psychiatric illness are particularly vulnerable to the types of epistemic injustice we have described above. In this section we discuss two key reasons for why epistemic injustice will likely be a feature of digital phenotyping in the psychiatric context: first, patients of psychiatry can be subject to particularly severe credibility deficits; and second, excess importance is afforded to scientific data compared to the patient’s lived experience. In short, we argue that the threat of epistemic injustice looms large for patients with a history of psychiatric illness who wish to challenge inferences made about their psychological states from digital data. Further, we briefly outline the potential harms that can arise as a result.

We have shown how certain marginalised groups are subject to credibility deficits in a medical context. We suggest that these credibility deficits are likely to be more severe in cases where the person has a history of, or concurrent, psychiatric diagnosis. Negative stereotypes of people with mental illness can often affect how patient testimony is evaluated by a clinician (McCradden et al. 2023). Patient testimony in psychiatric cases is often not rated as authoritative (Scrutton, 2017). Patients experiencing mental illness can be perceived to have a self-perception that is not reflective of realityPatient testimony in psychiatric cases is often not rated as authoritative as patients experiencing mental illness are often perceived to have a self-perception that is not reflective of reality (Coghlan and D’Alfonso 2021). This may be particularly applicable to people who have a history of delusions which involve an altered sense of reality. Empirical research has shown that patients are dismissed as lacking insight into their illness where they contradict decisions made by the treating clinicians. The patient’s denial of the illness is taken to be a manifestation of the illness itself, as is a patient’s refusal or reluctance to take medication (Nyttingnes et al. 2016). Scrutton (2017) suggests this may be particularly acute for diagnoses involving psychosis because the symptoms of psychosis can include delusions which would result in a legitimate loss of credibility. As Scrutton argues, mental health diagnoses can be particularly ‘sticky’: a past diagnosis results in confirmation bias that leads to ongoing testimonial injustice.

Paul Chrichton and colleagues provide an example from Crichton’s experience as a medical student where a young man in an inpatient unit in Munich claimed to be the relative of the then Soviet leader. His testimony was dismissed by the treating psychiatrist as a delusion and evidence of psychosis; it turned out that the patient was in fact related to the Soviet leader (Crichton et al. 2017). Scrutton (2017) also describes an example from Richard Bentall’s book: despite not reporting any pathological experience and no evidence of irrational behaviour, a person, Andrew, with a history of psychosis was detained on a psychiatric ward as a result of his “excessively polite” behaviour and decision to wear a suit to his grandmother’s funeral, which was considered a marker of grandiose behaviour (Bentall 2010, 111–112). In clinical practice, several empirical studies have demonstrated that psychiatric patients’ testimony is frequently dismissed by healthcare professionals in care settings, particularly in relation to patients’ medication preferences (Gustafsson et al. 2014; Nyttingnes et al. 2016; Ridley and Hunter 2013). Psychiatric patients are often not viewed as credible knowers about their own subjective experience of mental illness, or of the things that are beneficial or harmful to them such as medication and its side effects. In all studies, patients describe cases of having their concerns about medication side effects ignored: “I’ve asked what the side effects are and nobody will tell me ... The pills are upsetting my whole system, making me really ill. But no, it’s ‘take these pills because you’ve got this mental illness and everybody who’s got this mental illness has to have this pill’” (Ridley and Hunter 2013, 515). In some cases, patients had their advance directives (written while they were well) ignored by treating staff (Ridley and Hunter 2013). In a different study, nurses have described situations where patients’ somatic complaints are dismissed as manifestations of their mental illness being caused by delusions (Gustafsson et al. 2014).

Digital phenotyping for the detection of mental illness risks replicating these injustices. Consider a case where certain psychological states are inferred from a person’s digital phenotype and the person disagrees with the inference. Others have argued that all individuals are vulnerable to epistemic harms in such cases due to the sheer opacity of the data science technologies that produce inferences; rarely will either patients or clinicians have any insight into how various inferences have been arrived at from data inputs (Symons and Alvarado 2022; McCradden et al. 2023). No one but perhaps a very small number of data science experts will be able to scrutinise the algorithms, much of which may be legally protected from such scrutiny as proprietary trade secrets in any case (Symons and Alvarado 2022, 87). However, we argue that people with a history of psychiatric illness are even more vulnerable, as clinicians may be more likely to dismiss or disbelieve their testimony. Indeed, the person’s disavowal of the result of digital phenotyping may itself be taken as a symptom of the psychiatric illness. This may lead to a misdiagnosis and substandard medical care. In some cases, this may trigger some form of (unjustified) involuntary detention or treatment, as was the case with Andrew. The severe credibility deficit experienced by most patients with a history of mental illness is compounded by the credibility excess typically afforded to medical professionals, which only bolsters their confidence in their own assessment of the correct diagnosis.

This low credibility afforded to people with a psychiatric diagnosis may be compounded by a second feature of patient-clinician interactions in the healthcare system: the priority given to ‘objective’ or scientific data. Unlike most other areas of medicine, there are few ‘objective’ data that can be used to affirm or dispute the existence of a mental illness; clinicians cannot run a blood test or a scan to indicate whether someone is currently experiencing depression or schizophrenia. Instead, psychiatric diagnosis relies on a variety of behavioural symptoms, and clinical interviews between the patient and clinician. Patient testimony is central to the psychiatrist gleaning a medical history and determining the existence and severity of any symptoms. Given what is widely assumed to be the inherent unreliability of patient testimony in psychiatric cases, we consider it likely in many cases that clinicians will inflate the reliability and importance of digital data for promising a more ‘objective’ set of indicators of mental illness.^1^ As we argued in Sect. 2, the enthusiasm for digital phenotyping speaks of the aspiration for what are hoped to be “more objective” ways of measuring mental illness and to rely less on patient testimony. The way digital phenotyping is being framed reinforces the idea that the data gleaned from these devices will be more objective and reliable sources of information about a person’s experience than a person’s own self report.^2^ Further, the epistemic resources used in medicine prioritise certain types of evidence, favouring that which is considered medically relevant by the clinician, often at the cost of the patient’s experiential evidence of what it is like to have a certain illness, or, to not have it. The authority afforded to medicine as a science and clinicians as experts in their specialism renders the medical perspective the authoritative, and often exclusive, interpretation of the person’s experience (Buchman et al. 2017; Scrutton 2017).

There will undoubtedly arise numerous cases where even a clinically validated digital phenotyping tool reports a result that the patient will disagree with.^3^ It is important to acknowledge the real threat of epistemic injustice in these scenarios can also result in harms to patients (McCradden et al. 2023). Others have shown how digital information systems have influenced clinical decisions in the dispensing of prescription drugs: information provided by automated prescription drug monitoring programs has been shown to hold more weight in clinicians’ decisions to (not) prescribe than patients’ own testimony (Haines et al. 2022; Pozzi 2023). In some cases, this may lead to the denial of appropriate pain medication for patients. Similarly, we suggest clinicians relying too heavily on digital phenotyping at the expense of patient testimony could result in an erroneous clinical diagnosis or an unjustified involuntary detention or treatment order for psychiatric illnesses (McCradden et al. 2023). This will have obvious implications for patients who may therefore receive unnecessary or sub-therapeutic medical care. Others have also argued the inverse whereby a patient’s explicit request for help in an emergency department during a mental health crisis is dismissed in favour of an algorithmic prediction that rates them as low likelihood of in need of acute care, and they are refused treatment (McCradden et al. 2023).

Even more concerning is the possibility that digital phenotyping may result in treatment or detention decisions where clinicians feel they do not need to listen to a patient’s testimony at all. This is a particular worry across any country that uses an involuntary mental health treatment and detention regime. Decisions could be made to involuntarily detain or treat patients based on the data gleaned from digital sensing alone. While most clinicians would presumably not ignore patient testimony altogether, the perception that digital phenotyping offers an objective and hence more reliable view on whether a person is experiencing mental illness may offer a level of legitimacy in at least some instances to ignoring patient testimony and the patient’s interpretation of their own experience. In healthcare systems that are constantly striving for lower cost and more efficient healthcare, we should be alert to this risk. We should therefore be cautious about introducing digital phenotyping as a mechanism which has the potential to result in misdiagnosis, substandard care or an unjustified detention or treatment order.

A further, upstream harm relates to the likelihood that patients of psychiatry will have little opportunity to contribute to the development of the very resources used to make inferences about them. Previously, we described how patients are often unable to contribute to the reorientation of the hermeneutical resources used within medicine so that they take account of their lived experiences and concerns. Scrutton (2017) explains that the type of testimony sought from patients of psychiatry is pre-determined by the structure of the clinical psychiatric interview and the diagnostic categories of mental illness. In essence, insofar as patient testimony is sought, patients can be required to fit their testimony into an existing diagnostic categories. Scrutton (2017) suggests this leaves little room for the patient’s interpretation of their illness. As an example, Scrutton (2017) describes Holly’s experience of reporting hearing voices to her psychiatrist who sent her for EEG tests and told her she was hallucinating. Holly felt her psychiatrist hadn’t listened to what she had said about an experience that was positive and meaningful to her. Holly’s perspective is hermeneutically marginalised, which blocks a richer interpretation of her experience and possibly a more nuanced therapeutic response. There is often injustice when patients have alternative hermeneutical resources for understanding their experiences but there is a wilful refusal on behalf of the clinician (and medicine in general) to accept the patient’s interpretation as a supplement or even alternative to the biomedical one. It is problematic, Scrutton (2017) argues, to define and interpret the experiences of people with mental illness purely in third-personal terms without the inclusion of the subjective experience of the patient. The consequences of such a refusal to entertain alternative hermeneutical resources, or to include people who have particular experiences in the development and revision of epistemic resources for understanding those experiences, can result in a loss of crucial knowledge and the possibility of different or more nuanced treatment options.

Digital phenotyping risks replicating this hermeneutical injustice given its framing as an objective, third-person interpretation of a person’s psychological state. Currently, very few people with lived experience are involved in the development of digital technologies for mental health detection and diagnosis (Gooding and Kariotis 2021). In this recent scoping review, only four of 132 empirical research papers using algorithmic and data-driven technologies in mental health care included people with lived experience in a substantial way in the design, evaluation or implementation of the tools (Gooding and Kariotis 2021). It is therefore likely that alternative hermeneutical resources used for understanding mental illness, such as Holly’s, are omitted in the development of these tools. Relying solely on the dominant biomedical understanding of mental illness in the development of digital phenotyping means that these tools may miss important aspects of mental illness for patients that are worth monitoring, and important considerations in how or when tools should be used.

## Epistemic justice in digital phenotyping for psychiatry

Digital phenotyping is likely to advance in sophistication such that we eventually have clinically validated tools for detecting the presence of mental illness. Such tools will ideally facilitate earlier detection and treatment of illness and be of great benefit to patients and to healthcare systems. For example, tools such as those developed by Henson and colleagues may eventually enable an individual to self-manage their risk of schizophrenia relapse by providing them with prompts or notifications when their day-to-day patterns are diverging from their usual behaviours and asking them to consider checking in with a clinician or suggesting an appropriate intervention. If these tools are able to monitor a patient’s journey through an episode of mental illness and recovery, they may be useful in providing information about how well a certain medication is or is not working for a specific patient that bolsters patient testimony. This would go some way to countering some of the concerns, for example, raised by patients in the Ridley and Hunter study outlined in Sect. 4. Moreover, digital phenotyping may also be helpful in challenging clinician assumptions or biases about the presentation of mental illness in individual patients by providing data that may be relevant to that individual patient’s illness and testimony.

These benefits notwithstanding, the very nature of digital phenotyping renders it highly likely to be a site of epistemic injustice. In short, sometimes digital sensing will deliver the correct result, despite patient testimony to the contrary. At other times it will deliver a false or merely partial picture at odds with patient testimony, in which case the effects of epistemic injustice will lead to substandard care of individuals, both as epistemic agents and as patients of psychiatry.

The obvious question that arises then, is how to design and use digital phenotyping tools for the good, and to avoid the bad. For digital phenotyping to avoid perpetuating existing practices of epistemic injustice in psychiatric practice, the development and implementation of these tools for the detection of mental illness must attend to the risks we have outlined. While we do not aim to provide a comprehensive blueprint here, we conclude by suggesting the kinds of changes that would be needed to minimise epistemic injustice.

First and foremost, and as others have argued, recognising that patients have epistemically privileged first-person understandings of *what it is like* to have a certain illness or disability is integral to overcoming epistemic injustice is healthcare contexts (Carel and Kidd 2014; Scrutton 2017; Scully 2019). People with psychiatric conditions equally have epistemically valuable knowledge about the experience of treatment regimes.^4^ By attending to the accounts such people provide about their experiences of illness and treatment regimes, we gain a much better understanding of both. Scrutton cites accounts given by people with depression about experiencing an altered sense of time, diminished free will and altered bodily experiences. These are characteristics that are often not captured by the medical literature on depression (Scrutton 2017, 351). Alternative understandings of hearing voices and having visions, which are commonly associated with being symptoms of schizophrenia, can also be found by individuals in the Hearing Voices Network. Here, individuals explore rich and alternative understandings of voices. For example, voices or visions may be associated with trauma, a special gift, a spiritual experience or emotional distress (Hearing Voices Network 2023). For some, these experiences may be positive and bring value to an individual’s life. Sometimes these interpretations are not compatible with a formal diagnosis of schizophrenia or psychosis; but often they are. Even when there is no conflict between the medical diagnosis and the patient’s perspective, the patient’s knowledge can and should contribute significantly into how the illness is characterised and which treatment interventions, if any, are optimal given this patient’s own experience.

Certain consequences follow from taking patient testimony and hermeneutical resources seriously. Firstly, more care needs to be taken with the way digital phenotyping is framed. Rather than perceiving it as “the objective” and therefore authoritative indicator of a mental illness, we should treat clinically validated tools as one of a number of epistemic resources we can use to diagnose and treat mental illness. In particular, patient testimony must never be dispensed with or automatically distrusted just because it conflicts with the data gained from digital sensing. It should not be assumed that the knowledge gleaned from digital phenotyping is epistemically superior to the knowledge gained from listening to patients’ testimony and the frameworks they use for understanding their own illness. As Melissa McCradden and colleagues have argued, patient testimony must be actively sought and considered alongside AI tools to avoid the privileging of AI in clinical judgements or the reinforcement of power hierarchies between patients and clinicians (McCradden et al. 2023). Results from clinically validated digital sensing tools should at most trigger a conversation with the person. They should never alone be used to justify involuntary detention or treatment. It follows that some of the hype around digital phenotyping needs to be not merely tempered, but actively challenged on the basis that the way it is currently framed heightens the likelihood it will be used to further epistemic injustice.

Relatedly, we suggest that when digital phenotyping tools are being developed for the detection of mental illness, people with lived experience of that mental illness should be included in the design and development of the tool. As we have argued throughout this paper, patients of psychiatry have their own rich hermeneutical resources for understanding their mental illness. Given their expertise on what it is like to have a mental illness and their expertise of various treatment regimes, they will have valuable knowledge not only of what might be worth monitoring, but of *how* they want monitoring to happen, if and when it is to happen. Recent research has demonstrated that very few people with experience of mental illness are currently included in the design or deployment of digital mental health tools (Gooding and Kariotis 2021). Including people with lived experience from the outset can help ensure that the development of digital sensing tools incorporate a fuller understanding of what happens when a person is experiencing mental illness or its onset and can help guard against their improper use.

## Conclusion

We have argued that there are features of digital phenotyping which have the potential to render it a likely site of epistemic injustice in psychiatry. We described how epistemic injustice is common in the health and disability care context, with a focus on the credibility deficit experienced by patients in relation to clinicians when providing testimony, and the hermeneutical marginalisation of the epistemic resources they use to make sense of their illness or disability. We suggest that patients of psychiatry are particularly vulnerable to these types of epistemic injustice because of severe credibility deficits and the excess importance placed on ‘objective’ medicalised data in comparison to subjective reports of their lived experience. As a result, we argued that patients of psychiatry are vulnerable to epistemic injustice when digital phenotyping and patient testimony disagree. Such injustice has the potential to result in misdiagnosis, substandard medical care, or unjustified treatment or detention.

To counter the likelihood of epistemic injustice occurring in these contexts, we tentatively suggest changes that would minimise epistemic injustice in the design and use of digital phenotyping. First, we argued patients of psychiatry should be acknowledged as having equally valuable knowledge about what it is like to have a certain mental illness that can be used to gain a richer understanding of the illness itself and lead to better diagnosis, treatment and outcomes for patients. As such, we should reframe the way we view digital phenotyping as one of many epistemic tools available to us, rather than a uniquely objective tool for the diagnosis and treatment of mental illness. Digital phenotyping should at most trigger a conversation with a person; it should never alone be used to justify involuntary detention or treatment. Further, patients of psychiatry should be involved in the design and development of digital phenotyping from the outset, including advising on *how*, if and when these tools should be used, in order to help guard against their improper use.

## Footnotes

1 Others have argued that the mere absence of ‘objective’ markers can itself diminish a person’s credibility, such as for those suffering chronic pain. See Buchman et al. (2017) and Pozzi (2023).

2 Some suggest the perception of Artificial Intelligence (AI) as a superior form of knowledge may result in AI predictions being given more epistemic weight than both clinicians and patients. See McCradden et al. (2023).

3 Of course, there will likely also be cases where the clinician disagrees with the result of the digital phenotyping tool which may challenge the ?objectivity? of digital phenotyping on different grounds to the argument we pursue here. Such a scenario is beyond the scope of this paper, but we thank an anonymous reviewer for raising this point.

4 It is worth noting that claims about privileged first-person knowledge about what it is like to have a mental illness and what it is like to experience mental health treatment can be supported by, but do not depend on the truth of, standpoint theory (cf. Hartsock (1983), Harding (1986) and Collins (1990). The claim can also be supported by the very uncontroversial notion that one can acquire knowledge from experience, or, as is common to say more recently, ‘lived experience’.
